# Supplementary material for: New Histoprognostic Factors to Consider for the Staging of Colon Cancers: Tumor Deposits, Invasive Tumor Infiltration and High-Grade Budding
Source: Int J Mol Sci. 2023 Feb 10;24(4):3573. doi: 10.3390/ijms24043573 (PMC9959523; doi:10.3390/ijms24043573)
Supplement: Supplementary file 1 [file ijms-24-03573-s001.zip › ijms-2106930-supplementary.pdf]

Supplementary Material

Figure S1. Prognostic factors studied and overall survival and recurrence-free survival in 229 patients with colon cancer. (**A**) Tumor deposit impact on recurrence-free survival in patients with colon cancer. (**B**) Impact of tumor front invasion on recurrence-free survival in patients with colon cancer. (**C**) Budding impact on overall survival in patients with colon cancer.

A.

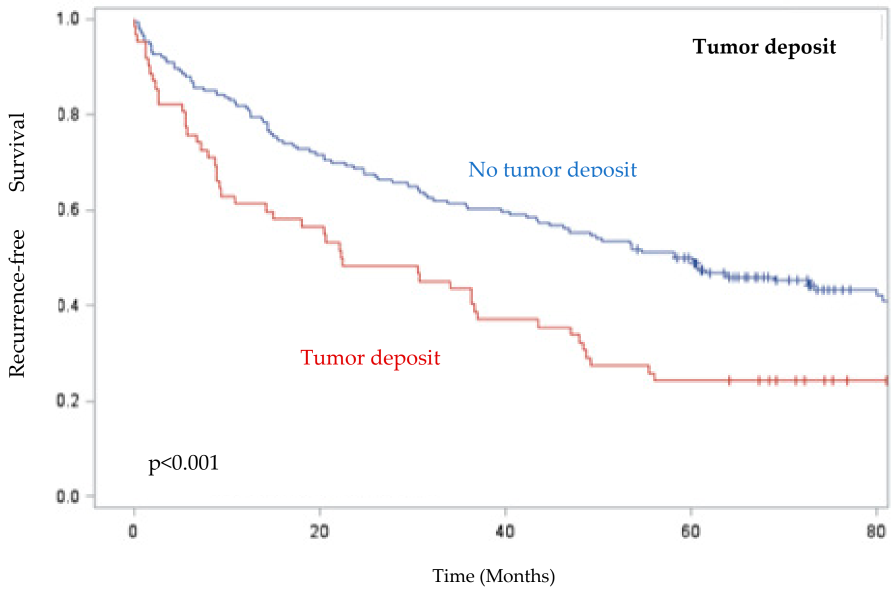

B.

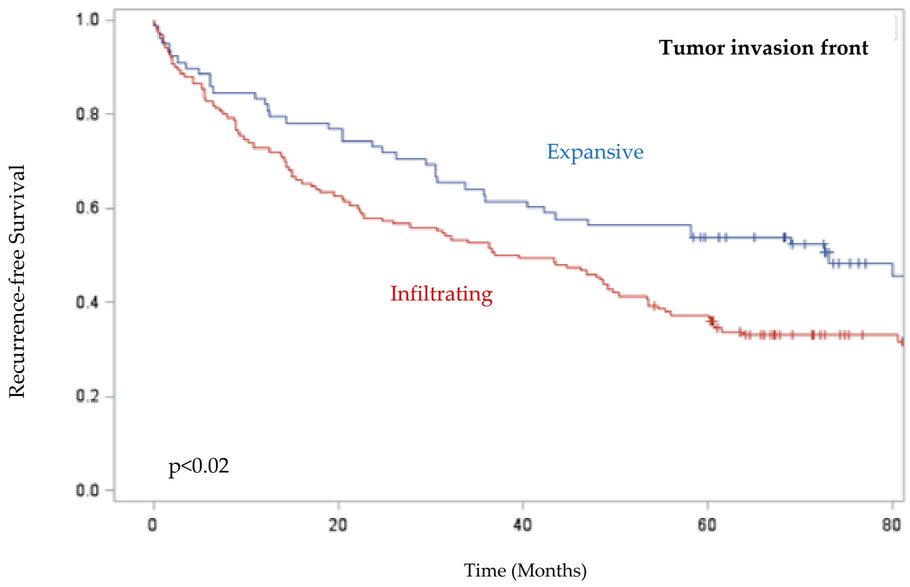

C.

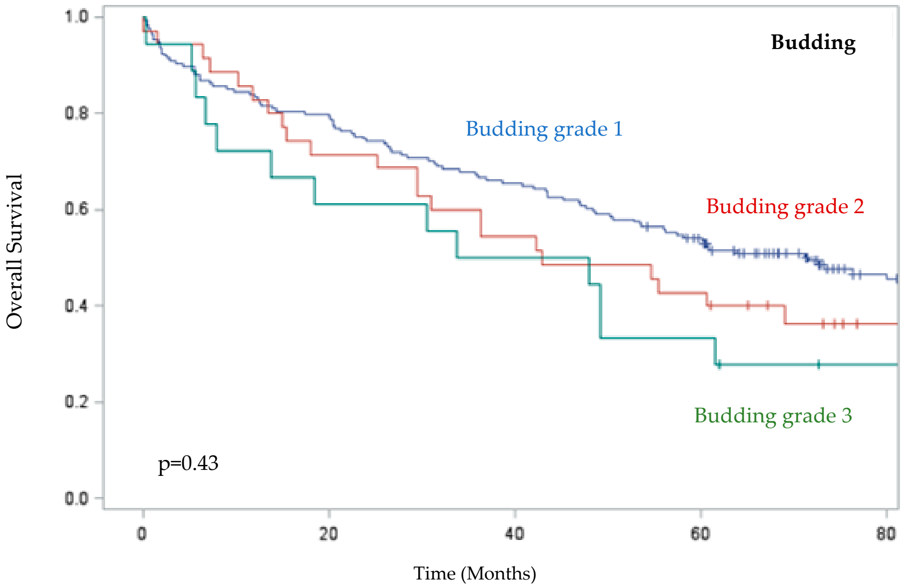

Budding
Budding grade 1
Budding grade 2
Budding grade 3
p=0.43
Overall Survival
1.0
0.8
0.6
0.4
0.2
0.0
0
20
40
60
80
Time (Months)
